# Long-Term Vaptan Treatment of Idiopathic SIADH in an Octogenarian

**DOI:** 10.3390/jcm6030028

**Published:** 2017-03-08

**Authors:** Stefan Büttner, Jürgen Bachmann, Helmut Geiger, Nicholas Obermüller

**Affiliations:** 1 Medical Clinic III, Department of Nephrology, University Hospital Frankfurt, Goethe-University, Theodor-Stern-Kai 7, 60590 Frankfurt, Germany; stefan.buettner@kgu.de (S.B.); helmut.geiger@kgu.de (H.G.); 2 Nephrologisches Zentrum Sauerland, Dialyse und Praxis Arnsberg-Hüsten, 59759 Arnsberg, Germany; bachmann@dialyse-arnsberg.de

**Keywords:** hyponatremia, SIADH, tolvaptan, elderly

## Abstract

Hyponatremia is the most common and by far underestimated electrolyte disorder in clinical practice. Especially in elderly patients, treatment of symptomatic hyponatremia is challenging. Herein we describe the case of an octogenarian with recurrent symptomatic hyponatremia due to idiopathic syndrome of inappropriate antidiuretic hormone release (SIADH). Fluid restriction was insufficient to prevent repeated episodes of hyponatremia complicated by falls and coma. After introduction of a low-dose therapy with tolvaptan, serum sodium levels as well as the clinical condition were stable under vaptan therapy, without any relapse for more than six years now. This case demonstrates that long-term tolvaptan treatment for hyponatremia caused by SIADH is safe and well tolerated, even in the elderly.

## 1. Introduction

Hyponatremia is the most common electrolyte disorder in clinical practice [1] and the incidence of hyponatremia increases with age [2]. Hyponatremia is associated with an increased rate of hospital admission, prolonged hospital stays [3] and is an independent risk factor for morbidity and mortality [2]. Mild hyponatremia leads to attention deficits, gait uncertainties, and falls [4], and represents a marker of frailty and an independent predictor of mortality in the elderly [5]. Treatment of hyponatremia caused by the syndrome of inappropriate antidiuretic hormone secretion (SIADH) can be challenging. The treatment of choice is restriction of daily fluid intake. Especially in the elderly, dehydration is a potential side effect of fluid restriction and it is not favorable in elderly patients who may have a low daily fluid intake. Vaptans are selective vasopressin receptor antagonists and exert their effects via the V2 receptor on renal principal cells. As “aquaretics”, they increase the output of electrolyte-free water and are approved for the treatment of SIADH [6]. In particular, geriatric patients with SIADH could benefit from treatment with vaptans. However, tolerance, efficacy, risks and benefits of long-term treatment have not been investigated in this special population. Herein we report the successful long-term treatment of an idiopathic SIADH with low-dose tolvaptan therapy in an octogenarian, who has had no relapse in more than six years.

## 2. Case Report

### 2.1. Patient History

In 2010, an 80-year-old female patient (Body Mass Index, BMI, 24.4 kg/m^2^) was admitted to our hospital for further diagnosis and treatment of recurrent, symptomatic hyponatremia. She reported recurrent syncopations in the two years prior to admission. The history included repetitive hospital admissions with intensive care treatment due to severe hyponatremia, with the last episode (106 mmol/L) in February 2009. Unfortunately, a clear diagnosis had not been established up to the date of admission. At that time, an ambulant control revealed a decreased plasma osmolality (252 mosmol/L) in combination with a urine osmolality of 367 mosm/L. Severe complications following the falls, such as bone fractures or traumatic bleeding, were absent. She underwent hip replacement because of arthrosis one year prior and her regular medication consisted of acetylsalicylic acid, amlodipine, and ezetimibe. A diuretic agent, particularly a thiazide diuretic, was not prescribed. In the months prior to hospitalization, restriction of fluid intake and administration of saline tablets was attempted. Moreover, a therapeutic approach with fludrocortisone, with the assumption of coexisting adrenal insufficiency, had no effect.

### 2.2. Inpatient Diagnostic Work-Up and Treatment Course

Clinical examination was unremarkable, especially without edema. Vital signs were stable. An ultrasound examination of the inferior vena cava showed a width of 18 mm and nearly complete collapse to 5 mm after inspiration. Laboratory data showed hypoosmolaric hyponatremia (serum sodium: 132 mmL/L; osmolality 264 mosmol/L). The urine osmolarity was 225 mmosmol/L and urine -sodium was 76 mosmol/L. The serum uric acid level was 4.3 mg/dL (reference: <5.7 mg/dL). Serum amino-transferases were within the normal range. Complete blood count, thyroid function (thyroid stimulating hormone (TSH), fT3 and fT4 within the normal range) and kidney function were normal without any signs of existing renal disease (serum creatinine 1.1 mg/dL, proteinuria < 140 mg/24 h). Further evaluation excluded adrenal insufficiency (aldosterone 13 pg/mL, cortisol 16 µg/dL, adrenocorticotropic hormone (ACTH) test normal). Imaging studies, including computed tomography of the head, chest and abdomen, excluded tumor growth. Additionally, echocardiography revealed normal heart function without any signs of valve disease. Previous investigations already excluded both renal and enteral salt wasting syndromes. Based on euvolemic hyponatremia and thorough exclusion of other causes, a diagnosis of idiopathic SIADH was established.

**Figure 1:**
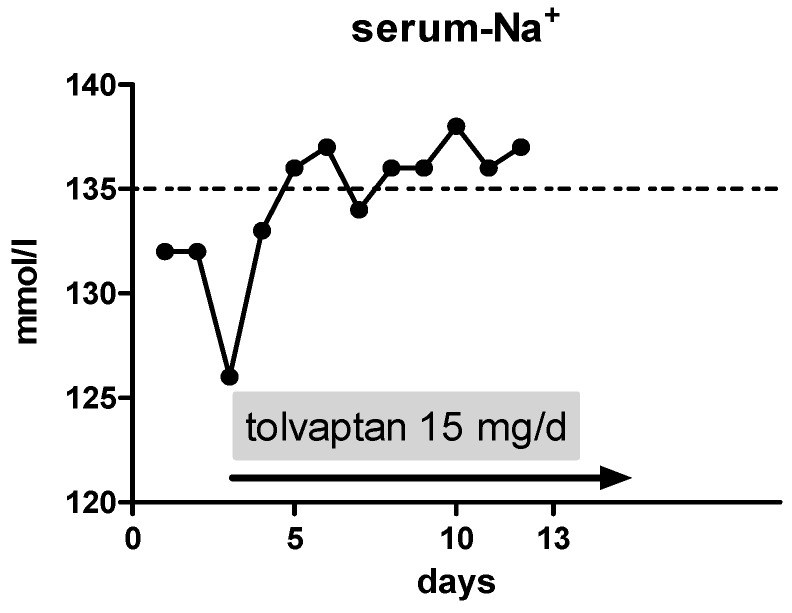
Course of serum sodium levels during the initial hospital stay: Tolvaptan was initiated with a daily dose of 15 mg on day 3. Serum sodium level at this point was 126 mmol/L. The line at 135 marking the lower limit of serum sodium levels by the local laboratory.

On day 3, we initiated an oral therapy with tolvaptan at a dose of 15 mg/day with liberalization of fluid restriction. Thereafter, the serum sodium levels climbed to 137 mmol/L (Figure 1), without any adverse event. At discharge, the dose of tolvaptan was still 15 mg/day.

### 2.3. Course over Six Years

**Figure 2:**
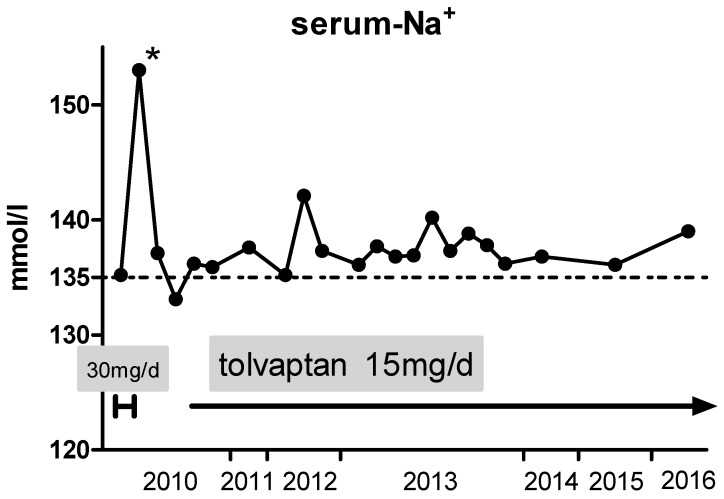
Six-year course of serum sodium levels after discharge from University Hospital Frankfurt: Administering a constant dose of 15 mg tolvaptan daily over the past five years, serum sodium levels remained within the normal range. The line at 135 marking the lower limit of serum sodium levels by the local laboratory. * Episode of hypernatremia following an increase of tolvaptan dosage to 30 mg per day in an external hospital (for details see text).

Ten days after discharge, admission to another hospital due to acute supraventricular tachycardia was necessary. After establishing sinus rhythm by electrical cardioversion, the colleagues initiated a treatment with dronedarone. However, without consulting the treating nephrologist, they also increased the daily dose of tolvaptan to 30 mg per day (Figure 2). Consequently, hypernatremia (153 mmol/L) occurred, which was followed by discontinuation of tolvaptan by the cardiologists. Furthermore, treatment with saline tablets and fluid restriction was re-initiated. At discharge, serum sodium levels dropped to 132 mmol/L. In the following outpatient care, the formerly established tolvaptan dose was re-initiated and serum sodium levels normalized.

The further course was uneventful and regular electrolyte controls remained unremarkable. Because of severe arthritis, she underwent total endoprosthesis of both knees. Tolvaptan therapy was not paused during the peri-operative phase and both interventions were without any periods of electrolyte imbalance or other complications.

Thereafter, her general condition was good. Dizziness or syncopes did not occur any more. Elektrocardiogram (ECG) monitoring documented a stable sinus rhythm under an antiarrhythmic therapy with dronedarone. She did not report any specific side effects of the vaptan therapy. The liver enzyme profile and parameters of liver synthesis were constantly within the normal range. Ambulatory blood pressure was also unremarkable. In the following years, heart ultrasound was performed regularly, documenting normal contractility of the left ventricle, accompanied by mild hypertrophy of the interventricular septum and moderate left atrial enlargement. Through 2015, severe aortic valve stenosis developed and the patient successfully underwent transcatheter aortic valve replacement in February 2016 with good functioning of the mechanical valve. Furthermore, kidney function declined, steadily leading to chronic kidney disease (eGFR/MDRD 41 mL/min).

Besides tolvaptan, regular medication includes clopidogrel (for six months after valve implantation), apixaban, pantoprazole, ramipril, torasemide and dronedarone. Serum sodium levels have remained stable in the lower reference range (Figure 2) for more than six years now. A further episode of life-threatening hyponatremia did not occur since the initiation of oral therapy with tolvaptan.

## 3. Discussion

Hyponatremia represents the most common electrolyte disorder in daily practice. Clinical manifestations range from apparently asymptomatic hyponatremia to life-threatening comatose states complicated by seizures. In particular, geriatric patients often reveal only minor symptoms, which might lead to missed diagnosis. At the same time, this cohort is at high risk for complications, such as gait disorders, reduced responsiveness, and concentration disabilities [4] which increase the risk of falls [4,7] and fractures [8]. Moreover, repeated admissions due to hyponatremia are associated with an increased mortality [9].

The severity of clinical symptoms as well as the underlying pathophysiology should guide the therapeutic approach. Treatment of acute, severe hyponatremia with neurologic symptoms consists of administration of hypertonic (3%) saline solution until neurologic symptoms subside. Close electrolyte monitoring under intensive care settings and awareness to avoid inadvertent overcorrection is mandatory in such cases. After treating this life-threatening situation, a causal therapy should follow [10]. Particularly in patients older than 70 years, there is often more than one underlying cause for hyponatremia and a careful assessment is mandatory. At least in part, disturbances in sodium handling are due to age-related changes in the physiological adaptation of water and electrolyte balance. In addition, infections, malignancies and various medications can trigger and aggravate hyponatremia. While establishing the differential diagnosis of hyponatremia, it is essential to rule out these underlying causes.

The most frequent causes for chronic hyponatremia in the elderly are the use of thiazide diuretics [11], hypovolemia, heart failure and SIADH [5,9,12]. Measurement of antidiuretic hormone (ADH) is not helpful to differentiate these causes. The more stable copeptin, also called C-terminal proarginine vasopressin, might be an interesting marker to differentiate SIADH from other hyponatremic states [13]. The treatment of choice in the case of hyponatremia caused by SIADH is fluid restriction. Urea is recommended as an alternative [10], but, because of the bitter taste of urea, might not be tolerated by the vast majority of patients. Salt tablet administration, in particular in older patients, is futile, and salt intake may increase the total amount of sodium chloride in the body, and therefore may worsen hypertension and heart insufficiency. If fluid restriction fails, initiation of the oral selective vasopressin V2-receptor antagonist is an option.

Tolvaptan, approved for the treatment of euvolemic hyponatremia in Europe, was shown to be effective with good tolerability [14]. In that long-term investigation (SALTWATER trial, mean follow up time 1.9 years per patient), tolvaptan was administered open-label to hyponatremic patients with euvolemia, heart failure or cirrhosis. The mean baseline serum sodium was 130.8 mmol/L and increased >135 mmol/L throughout the study period, with a mean tolvaptan dosage of 30 mg/day. Importantly, mean age was 64.6 years and many patients suffered from severe underlying disease [14]. Preexisting fluid restriction must be relaxed due to fluid loss based on the “aquaretic” effects of vaptans. Additionally, it is necessary to avoid dehydration-promoting factors and patients should be encouraged to achieve a sufficient daily fluid intake. Acute and profound diarrhea should prompt pausing of tolvaptan and, in such cases, administration of intravenous supplementation of fluids might be necessary. On the other hand, non-critical increase of the tolvaptan dose might result in hypernatremia, as documented in the present case. It is advisable to interrupt or terminate a therapy with tolvaptan only after a thorough benefit-risk assessment. In the case of idiopathic SIADH, withdrawal from medication can provoke a rebound of hyponatremia. Vaptan therapy belongs in the hands of experienced physicians, providing safety and therapeutic efficacy as shown here.

A potential side effect of tolvaptan is an increase in liver enzymes, and therefore, regular examination of liver function is mandatory. However, liver enzyme levels return to baseline after withdrawal of tolvaptan [15,16]. Using tolvaptan at 15 mg per day as described in our case, there were no clinical or laboratory signs of hepatotoxicity even in the long term. Belonging to the manufacturer, there were no signs of hepatotoxicity for doses up to 60 mg/day in the entire post-approval clinical phase.

Another important point is the potential of interaction with drugs metabolized by Cytochrome 3A4 (CYP 3A4). CYP3A4 enzyme inhibition by other drugs or nutritional compounds can significantly increase tolvaptan blood levels. This might be problematic in elderly patients, where polypharmacotherapy is common. An aquaretic therapy does not exclude the simultaneous prescription of drugs influencing the volume and salt balance such as blockers of the renin-angiotensin system, but they should be applied in lower doses with close patient monitoring. However, the presented case shows that problems of everyday life do not hamper safe long-term therapy, even in the elderly.

## 4. Conclusions

The presented case shows that long-term treatment—for more than six years—of symptomatic hyponatremia due to idiopathic SIADH using tolvaptan is safe and feasible, even in the elderly.
